# Tracking the transcriptional host response from the acute to the regenerative phase of experimental pneumococcal meningitis

**DOI:** 10.1186/1471-2334-10-176

**Published:** 2010-06-17

**Authors:** Matthias Wittwer, Denis Grandgirard, Janine Rohrbach, Stephen L Leib

**Affiliations:** 1 Institute for Infectious Diseases, University of Bern, Friedbuehlstrasse 51, CH-3010 Bern, Switzerland

## Abstract

**Background:**

Despite the availability of effective antibiotic therapies, pneumococcal meningitis (PM) has a case fatality rate of up to 30% and causes neurological sequelae in up to half of the surviving patients. The underlying brain damage includes apoptosis of neurons in the hippocampus and necrosis in the cortex. Therapeutic options to reduce acute injury and to improve outcome from PM are severely limited.

With the aim to develop new therapies a number of pharmacologic interventions have been evaluated. However, the often unpredictable outcome of interventional studies suggests that the current concept of the pathophysiologic events during bacterial meningitis is fragmentary. The aim of this work is to describe the transcriptomic changes underlying the complex mechanisms of the host response to pneumococcal meningitis in a temporal and spatial context using a well characterized infant rat model.

**Methods:**

Eleven days old nursing Wistar rats were infected by direct intracisternal injection of 2 × 10^6^cfu/ml of *Streptococcus pneumoniae*. Animals were sacrificed at 1, 3, 10 and 26 days after infection, the brain harvested and the cortex and hippocampus were sampled. The first two time points represent the acute and sub-acute phase of bacterial meningitis, whereas the latter represent the recovery phase of the disease.

**Results:**

The major events in the regulation of the host response on a transcriptional level occur within the first 3 days after infection. Beyond this time, no differences in global gene expression in infected and control animals were detectable by microarray analysis. Whereas in the acute phase of the disease immunoregulatory processes prevail in the hippocampus and the cortex, we observed a strong activation of neurogenic processes in the hippocampal dentate gyrus, both by gene expression and immunohistology starting as early as 3 days after infection.

**Conclusions:**

Here we describe the cellular pathways involved in the host response to experimental pneumococcal meningitis in specified disease states and brain regions. With these results we hope to provide the scientific basis for the development of new treatment strategies which take the temporal aspects of the disease into account.

## Background

Bacterial meningitis (BM) is associated with a mortality rate of up to 30% and up to 50% of the surviving patients suffer from permanent neurological sequelae which include deafness, learning impairment, seizure disorders and cerebral palsy [1-3]. The most common etiological agent of non epidemic BM is *Streptococcus pneumoniae *(pneumococcus) [4]. Among the different forms of bacterial meningitis, pneumococcal meningitis is associated with the highest case fatality rate and incidence of neurological sequelae [1,5,6]. Morbidity and mortality have largely remained unchanged over the last decades in spite of advances in antimicrobial and intensive care therapies [7]. Therapeutic options to reduce acute injury and to improve recovery from BM are limited [8]. In BM, the only clinically used, adjunctive therapy is the administration of dexamethasone during the acute disease phase [2,8]. While this leads to improvement primarily on mortality in adult patients, there is currently no conclusive evidence that the drug is beneficial in paediatric patients [2,8,9]. Given the limited success in reducing brain damage during the acute disease, it appears imperative to expand the scope of strategies from the acute disease phase into the recovery phase with the aim to improve the outcome of brain injury. Thus, current therapies for BM are insufficient and new approaches to the adjunctive therapy of BM are needed. Understanding the processes of brain damage and repair following BM is a prerequisite for the development of new drugs that can preserve and restore neuronal function.

The aim of this work is to describe the transcriptomic changes underlying the complex mechanisms of the host response to pneumococcal meningitis in a temporal and spatial context. For this purpose we evaluated the gene expression profile of the two brain structures predominantly affected by brain damage, i.e. the cortex and the hippocampus, at four different stages of the disease in an infant rat model.

The continuously growing pool of biological metadata provides the possibility to shift the interpretation of transcriptomic data from a "gene by gene" approach to a more biological system-based analysis. In the present work we describe the transcriptomic data under two aspects: the categorization of regulated genes based on the defined and organism independent vocabularies of the Gene Ontology Database [10] and the Kyoto Encyclopedia of Genes and Genomes (KEGG) pathway database [11-13] a collection of graphical cellular pathway informations. Due to the complexity of the samples in regard to the different cell types within a given brain structure, association of transcriptional data with histomorphological findings is helpful in validating the findings. Therefore we assessed the presence and tissue distribution of two pathogenetically relevant cell types namely the microglia and proliferating neuronal stem/progenitor cells. The microglia plays a major role in the initiation and coordination of the innate immune response of the acute disease phase [14-18]. Microglia adopt an amoeboid phenotype and a phagocytic- and antigen-presenting-cell-type-function during the later phase [14-16,19]. The proliferation, differentiation and functional integration of neuronal stem/progenitor cells are a prerequisite for the repair of damaged tissue and the re-establishment of neurofunctional integrity. In this work we identified regulative pathways that influence tissue homeostasis in the different stages of the disease and which may provide new therapeutic targets for the reduction of neuronal sequelae.

## Methods

### Model of experimental bacterial meningitis

We used an established model of experimental pneumococcal meningitis in infant rats [20]. All animal studies were approved by the Animal Care and Experimentation Committee of the Canton of Bern, Switzerland, and followed the Ethical Principles and Guidelines for Experiments on Animals by the Federal Veterinary Office of Switzerland. On postnatal day 11, nursing Wistar rats (Charles River, Germany) were infected by direct intracisternal injection of 10 μl saline solution, containing 2 × 10^6 ^cfu/ml of *Streptococcus pneumoniae *(serogroup 3), with a 32 gauge needle. Sham-infected animals were injected with 10 μl of sterile pyrogen free saline solution. Eighteen hours after infection and at predetermined later time points, animals were weighted and clinically assessed using the following scale of scores: 1 = coma; 2 = does not turn upright after being placed on the back; 3 = turns upright within 30 s; 4 = minimal ambulatory activity, turns upright in less than 5 s; 5 = normal [20]. Animals with a score of 2 or below were immediately sacrificed for ethical reasons. Cerebrospinal fluid (CSF) was obtained by puncture of the cisterna magna and 5 μl were cultured quantitatively on agar plates in order to document successful infection. At the same time point antibiotic treatment of the infected animals with 100 mg/kg ceftriaxone (Roche Pharma, Reinach, Switzerland) subcutaneously (s.c.) was initiated. Cisternal puncture for CSF sampling was repeated at 1 and 3 days post infection. After withdrawal, CSF samples were immediately centrifuged for 10 min/4°C/13'000 rpm (10'000 g) and the supernatant was frozen at -80°C and stored for further analysis.

### Study design

Animals were sacrificed by an overdose of intraperitoneal (i.p) pentobarbital (100 mg/kg Abbot Laboratories, Chicago, USA) at the predefined time points e.g. 1 day, 3 days, 10 days and 26 days after infection. The first two time points represent the acute and the subacute disease-phase of bacterial meningitis respectively, whereas the latter two timepoints represent the recovery phase of the disease. At each time point 6 infected and 6 sham infected animals were sacrificed. Control and infected animals were age matched to avoid differences in the gene expression patterns due to developmental processes in the infant rat brain. Animals were perfused via the left cardiac ventricle with 30 ml of RNAse-free ice-cold phosphate-buffered saline (PBS). Immediately after brain removal, the meninges were removed and the brain was divided into the two hemispheres. From the left hemisphere the cortex and the hippocampus were dissected, transferred into RNAlater^® ^(Applied Biosystems/Ambion, Rotkreuz, Switzerland) and stored at 4°C until subsequent RNA extraction; the right hemisphere was fixed in paraformaldehyde (PFA) 4% in PBS for the subsequent histopathological assessment of brain injury.

### Tissue processing and histopathology

To assess the effect of bacterial meningitis on brain damage, the brains were evaluated histomorphologically. After sacrifice, animals were perfused with phosphate buffered saline (PBS), brains were removed and postfixed in PFA 4% in PBS overnight at 4°C. Subsequently the brain tissue was dehydrated in ascending alcohol and embedded in paraffin. From each brain four 10 μm sections of the dentate gyrus were cut and mounted on a glass slide and stained with cresyl violet (Nissl stain). Neurons in the dentate granule layer of the hippocampus with a nuclear morphology typical for apoptosis (condensed, fragmented nuclei and/or apoptotic bodies) were counted in 3 visual fields (magnification, × 400) in each of the 4 blades of the dentate gyrus applying the following scoring system: 0-5 cells = 0; 6-20 cells = 1 and >20 cells = 2. This scoring system has been previously validated by other methods of apoptosis detection including active caspase-3 and TUNEL staining [21].

### Immunohistochemistry

For microglia staining, 10 μm cryo-sections were mounted on chrom alum/gelatine-coated slides and incubated overnight with a monoclonal mouse anti-AIF-1 antibody (Iba-1, Wako Pure Chemical Industries, Ltd.) or with a monoclonal mouse anti-rat CD68 antibody (AbD Serotec GmbH, Düsseldorf; Germany). A goat anti-mouse IgG Cy3-conjugated antibody (Jackson ImmunoResearch Laboratories, West Grove, PA) was used as secondary antibody. The slides were mounted with Mowiol^® ^(Merck, Darmstadt, Germany) containing 2.5% Dabco^® ^(Sigma) and photographed with an Axiophot fluorescence microscope equipped with a digital camera.

### Bromodeoxyuridine labeling and histomorphometry

To document cell proliferation from the acute phase of the disease into the late phase, infant rats received 50 mg/kg BrdU (Sigma) at day 1 to 3 after infection. BrdU is a thymidine analog which incorporates into newly synthesized DNA [22,23].

Six micrometers thick brain sections were stained for incorporated BrdU (anti-BrdU, Dako Schweiz AG, Baar, Switzerland) and counterstained with 4',6-Diamidin-2'-phenylindol- dihydrochlorid (DAPI). For cell density counting, digitized images, acquired using a conventional microscope and a charge-coupled device camera, were used. Using ImageJ software (NIH), regions of interest were outlined in the dentate gyrus, using the DAPI-stained image and superimposed on the BrdU image. After calibration, the surface areas were determined and a cell proliferation determined as the number of BrdU positive cells/unit area [22].

### RNA processing, quality control and chip hybridization

Tissue samples of the cortex and the hippocampus from the 12 control and from the 12 infected animals were processed and analyzed separately; thus in total 48 arrays were hybridized. Total RNA was extracted from brain samples using RNeasy^® ^Lipid Tissue kit (QIAGEN, Basel, Switzerland) and purified with RNeasy columns (QIAGEN, Basel, Switzerland). Quantification and assessment of RNA integrity were performed on the Agilent 2100 bioanalyzer platform (RNA 6000 Nano, Agilent technologies, Waldbronn, Germany) and validated on the NanoDrop^® ^(NanoDrop, Wilmington, USA) quantification device. Based on RNA quality control results and histopathological evaluation of hippocampal damage (apoptosis score) RNA extracts from 3 infected and 3 control animals were selected for each time point for array hybridization. Double-stranded cDNAs were synthesized from 5 μg of total RNA using an oligo dT-T7 promoter primer (Roche Molecular Biochemicals, Mannheim, Germany). The cDNAs obtained were used as templates for in vitro transcription using the Megascript kit purchased from Ambion (Austin, TX) and biotinylated nucleotides (Bio-11-CTP and Bio-16-UTP) provided by Roche Molecular Biochemicals (Basel, Switzerland). Fragmented in vitro transcripts (cRNAs) were hybridized overnight onto commercially available rat microarrays (GeneChip^® ^Rat Genome 230 2.0 Array, Affymetrix, Santa Clara, CA) containing 31'000 probe sets representing approximately 28'000 well-substantiated rat genes. The hybridized samples were stained with streptavidin-R phycoerythrin (SAPE, Molecular Probes Inc., Eugene, OR) and the signal was amplified using a biotinylated goat anti-streptavidin antibody (Vector Laboratories, Burlingame, CA). Washing, staining and amplification were carried out in an Affymetrix GeneChip^® ^Fluidics Station 450. Microarrays were scanned in an Affymetrix GeneChip^® ^scanner 3000. Signal intensities were calculated based on image files with the Affymetrix GeneChip^® ^Operating software (GCOS) v1.1.1. Array data was deposited and is curated at the ArrayExpress platform http://www.ebi.ac.uk/microarray-as/ae/. The accession number is E-MEXP-1656.

### Datamining

Chip data analysis was performed on the R platform for statistical programming using packages of the Bioconductor project [24]. Due to the asymmetric distribution of microarray data all datasets were log2 transformed. Background correction, normalization and data summary were performed with non linear methods using the rma function of the affy package [25]. Chip quality assessment was exploratively performed based on boxplots of the raw log scale intensities and MA-plots visualizing signal intensity dependent effects on the log-ratios. Additionally the quality of the hybridized cRNA was assessed by RNA digestion plots where the mean intensities of all probes on an array are plotted according to their 5' to 3'probeset position (affy package).

To reduce the number of hypotheses to be tested in the subsequent significance tests, gene filtering was performed. All genes that were expressed under the estimated background intensity of 60 fluorescent units (FU) on at least 3 of the 48 chips and genes showing an interquarantile range (IRQ) of less than 0.5 were excluded from significance testing.

Significance testing was performed using the limma package [26,27]. Type 1 error was corrected by the Benjamini-Hochberg false discovery rate algorithm [28].

Gene ontology (GO) statistics was calculated with the GOstats package [29]. For each time point the according significantly regulated genes were associated to the biological process specific GO identifier. The representation of a given GO identifier in the population of the significant regulated genes was compared to the abundance of the same GO identifier in the total brain transcriptome. To test if a given GO identifier is significantly more abundant in the population of regulated genes a hyper geometric distribution was used to calculate p-values. This approach allows bringing a selected group of transcripts in a biological context by following the trace of significantly over-represented GO terms throughout the GO structure.

**Figure 1:**
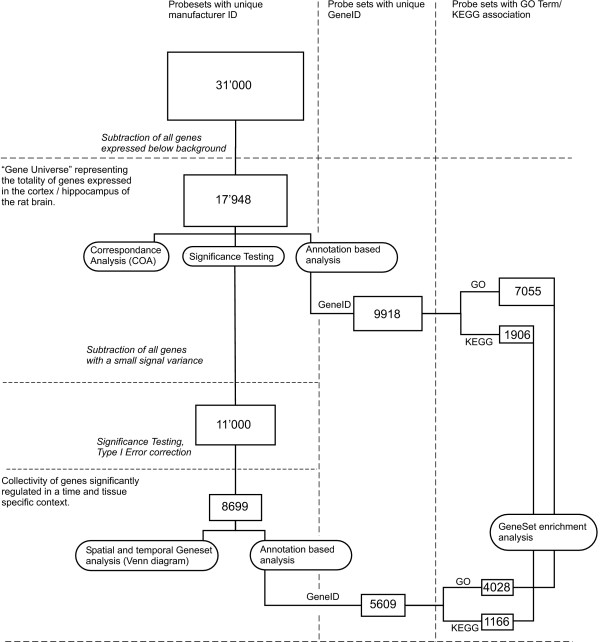
**Data Analysis Flowchart**. Figure 1: Overview of the data analysis strategy chosen for the present work. The numbers in the boxes indicate the size of the gene pool. Oval boxes describe the used data analysis/reduction strategies. The vertical dashed lines indicate the stepwise reduction of the gene set according to the available information of the metadata resources. The horizontal dashed lines indicate the reduction of the gene set based on statistical analysis.

Correspondence Analysis (COA) was carried out with the made4 [30] package which provides several algorithms for multivariate microarray data analysis. An overview of the data mining workflow is provided in Figure 1.

### Data Dimension Reduction: Correspondence Analysis (COA)

The aim of the diverse dimension reduction methods is, as the name implies, to reduce the essence of the interrelation of multidimensional data to a lower complexity. This reduction to few transformed variables allows the graphical interpretation of complex data in the two or three dimensional space. In contrast to principal component analysis (PCA) which is used for the exploratory description of linear trends in data (eigenanalysis of a covariance matrix), COA analyses the association between microarray samples and genes with a 2 way contingency table based chi-square statistics (eigenanalysis of a chi-square distance matrix). It has been shown, that compared to PCA, COA is less vulnerable to unimodal or non-linear trends which are often present in biological data. In our study COA analysis was performed on the median expression values of each experimental group.

### PCR

The SuperScript™ II First Strand cDNA Synthesis System (Invitrogen, Basel, Switzerland) was used with 5 μg total RNA and 250 ng random hexamer primers (Promega, Madison, WI), following the manufacturer's protocol. To minimize methodological effects interfering with cDNA quantification all total RNA samples were processed in parallel. The cDNA samples (CX 1.6 μg/μl; HC 1.1 μg/μl) were diluted 1 to 5 with RNAse-free H_2_O and aliquots (CX 0.3 μg/μl; HC 0.2 μg/μl) were stored at -20°C, respectively at -80°C. Template cDNA was amplified using the ABIPrism 7700 Sequence Detection system (Applied Biosystems, Foster, CA). For TGF-β1, Aquaporin 4 and Folate Receptor 2 amplification detection the SYBR-green PCR Master Mix (Qiagen, Basel, Switzerland) was used. The specificity of the SYBER-green signal was validated by the presence of a single sharply defined melting peak for every PCR run. The amount of template used for the PCR reaction was normalized to the commonly used housekeeper gene Glyceraldehyde-3-phosphate dehydrogenase (GAPDH). GAPDH quantification was performed with the TaqMan^® ^rodent GAPDH control reagents from Applied Biosystems (Foster City, CA) containing a VIC™ labeled GAPDH probe (excitation at 554 nm) and a forward and reverse primer set.

Oligonucleotide primers used in this study were designed with Primer Express™ software Version 2.0 (Applied Biosystems, Foster City, CA) and were supplied by Microsynth laboratories (Balgach, Switzerland). Primer sequences and GenBank association numbers are listed in table 1.

**Table 1:** PCR Primers and Results

Name	Forward	Reverse	Ratio Array	Ratio PCR
Folate receptor 2 (XM_215013)	GATTGCCCTGGGTTGAGTCA	TCACTCCTACAAGGTCAGCAACTATAG	41.7	22.3
Aquaporin 4 (NM_012825)	GCTTTCTTCTCCCAAGCACAA	TCTTGCTGCCACAGACACAAA	5.8	2.6
TGF-β (NM_021578)	GGAAGGGTCGGTTCATGTCA	CGTGGAAATCAATGGGATCAG	2.5	2.2

Primers used for the assessment of the expression level of the low abundant Folic acid receptor, the high abundant Aquaporin 4 water channel and the intermediately expressed growth factor TGF-β1.

### Luminex

The commercially available Rat 9-Plex cytokine panel A from BIO-RAD was used (BIO-RAD, Hercules, CA, kit# 171-K11070) to assess the protein levels of IL-1α/β, IL-2, IL-4, IL-6, IL-10, GM-CSF, IFNγ and TNFα with the Luminex^® ^xMAP™ technology. CSF samples were centrifuged and the supernatants were diluted 1:5 in Bio-Plex rat serum sample diluent and mixed with antibody-coated microspheres on a membrane microtiter plate. In the 9-plex kit, nine different microspheres with a specific spectral signature are included, each coated with an antibody specific for one cytokine. The bound cytokines were detected with a secondary antibody labeled with biotin. Signal was enhanced by a streptavidin/phycoerythrin complex. Flow cytometry and signal detection was performed by Bio-Plex™ 200 system and raw data were analyzed with Bio-Plex Manager 4.1 software.

Statistical analysis of protein concentration data was done with GraphPad prism version 4.0 (GraphPad Software Inc., San Diego, CA) by 1 way ANOVA. Pair wise group comparison was calculated by Tukey's multiple comparison test.

Protein quantification was performed using the colorimetric BCA Protein assay kit (Pierce, Rockford, IL). Quantification of the samples was based on a BSA calibration curve with ascending concentrations from 0 μg/ml to 2 μg/ml.

## Results

### Clinical parameters of meningitis

All rats infected with *S.pneumoniae *developed meningitis as documented by positive bacterial CSF cultures (log10 6.8 - log10 8.0 cfu/ml) at 18 h after infection, altered clinical score and reduced weight gain compared to uninfected animals (table 2) . Sham-infected animals (n = 24) did not show clinical signs of disease.

**Table 2:** Clinical Parameters

Clinical parameters				
**Time**	**Status**	**Weight**	**Clinical score**	**Apoptosis score**

1 day	Control	27.6 (100%)	5	0
	Infected	23.81 (86%)	3	1.66
3 days	Control	34.5 (100%)	5	-
	Infected	24.1 (69.8%)	4	-
10 days	Control	54.2 (100%)	5	-
	Infected	44.9 (82.2%)	5	-

### Apoptotic cells in the dentate gyrus

As reported previously, infection with *S.pneumoniae *causes apoptosis in the subgranular zone of the hippocampal dentate gyrus [20,31,32]. During the acute disease (1 day after infection) the median score of apoptotic neurons was 1.66. In the late acute phase of disease (3 days after infection) the number of apoptotic cells decreases to control levels [21]. In animals assessed 10 days and 26 days after infection, no apoptotic damage was detectable in the dentate gyrus. Apoptosis was exclusively localized to the subgranular zone of the hippocampal dentate gyrus. This observation is in agreement with previous findings that immature neurons in the subgranular zone are most prone to apoptosis [22].

### Inflammatory parameters

Nine different cyto-/chemokines were assessed on the protein level in CSF samples collected at 1 day after infection. The pro- inflammatory cytokines IFN-γ, TNF-α, IL-1α, IL-1β, IL-6 and IL-10 showed a significant up-regulation in infected animals compared to sham-infected animals. Levels of GM-CSF, IL-4 and IL-2 were not significantly altered in infected vs. sham-infected animals (table 3). Non-parametric spearman correlation of protein concentration ratios with the according gene expression ratios was highly significant (rs = 0.83; p = 0.0052).

**Table 3:** Luminex Results

	Cytokines [pg/ml]								
	**IFNgamma**	**TNFalpha**	**IL-1alpha**	**IL-1beta**	**IL-6**	**IL-10**	**GMCSF**	**IL-4**	**IL-2**

Control	141	401	38	170	142	108	172	83	452
Infected	5833	4418	623	11611	47199	2192	145	68	623

Ratio [inf/cont]	41.4	11.0	16.4	68.3	332.4	20.3	0.8	0.8	1.4

Assessment of 9 different chemo-/cytokines in the CSF of infected and uninfected rats 1 day post infection.

### PCR

To validate the microarray data the expression levels of 3 genes were assessed by real-time PCR. Chip sensitivity and signal linearity were estimated by selecting a set of genes which spans an expression range from 60 FU (background level) to 8000 FU.

Gene regulation was assessed for the low abundant Folic acid receptor, the high abundant Aquaporin 4 water channel and the intermediately expressed growth factor TGF-β1. The highest discrepancy in expression ratios between PCR and GeneChip quantification was seen for the low abundance transcript coding for the Folic acid receptor. This is most likely due to the low expression value of this transcript in the control animals which lies below the background level. Thus the lack of accurate expression estimates for the controls hampers the calculation of an expression ratio. This notion holds true for all transcripts with a below-background-expression in a given treatment group. Expression ratio calculations for intermediate and high abundance genes were similar in both methods.

The Pearson correlation of infected vs. control ratios between RT-PCR and GeneChip data was r = 0.998 (table 1).

### Microarrays

From totally 31'000 probe sets of the rat genome represented on the chip, about 17'000 were expressed over background level. Of these expressed probes a subgroup of 11'000 transcripts showed a substantial signal variation (IQR of >0.5) and thus were included in subsequent hypothesis testing. After statistical analysis 8699 probes were found to be significantly regulated in infected animals compared to sham-infected controls over all of the investigated time points and tissues.

An Entrez ID could be assigned to 5609 individual genes which were included in the subsequent gene annotation based statistical analysis (see Figure 1 for an overview of the data mining workflow).

### Spatial and Temporal Gene Expression

**Figure 2:**
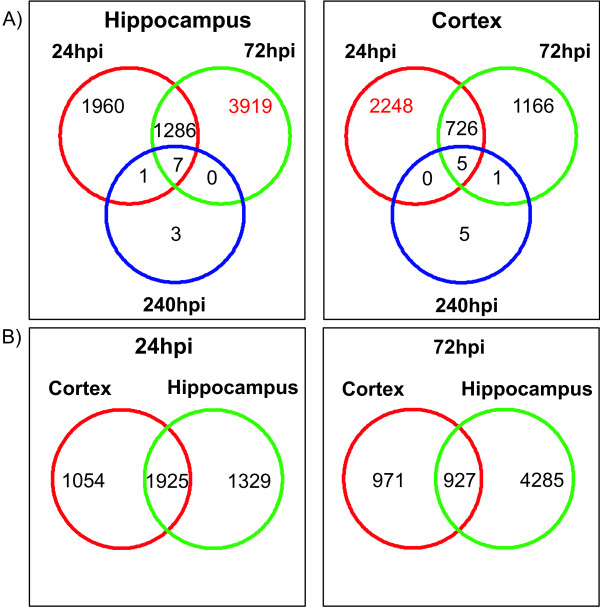
**Venn Diagrams**. Schematic of the spatial and temporal gene expression in the experimental groups. The numbers in the circles represent the number of transcripts which are significantly up- or down-regulated in a given condition. A) Comparison of the extent of transcriptional regulation within the cortex and the hippocampus in relation to the time after infection. In contrast to the cortex which shows the highest number of regulated genes at 1 day after infection, the transcriptional regulation in the hippocampus peaks 3 days after infection. B) In the early acute phase of the disease at 1 day after infection, the hippocampus and the cortex share ~60% of the significantly regulated transcripts. At 3 days after infection, in the late acute phase, the number of co-regulated genes drops to 50% for the cortex and 18% for the hippocampus.

In the cortex the peak of transcriptomic regulation in regard to the number of transcripts which are significantly regulated was observed at 1 day after infection (2974 transcripts). At 3 days after infection the number decreased by ~ 32% (1892). At 10 days after infection only 11 transcripts were found to be regulated (Figure 2A). At 26 days after infection there was no detectable difference between the gene expression profiles of infected and control animals.

In contrast to the cortex the peak of transcriptional regulation in the hippocampus was observed at 3 days after infection (5205 transcripts) with an increase of 32% compared to the acute phase at 1 day after infection of the disease (3246 transcripts). In accordance to the findings in the cortex the number of regulated transcripts drops to 11 at 10 days and 0 at 26 days after infection (Figure 2A). Assessment of the proportion of genes concomitantly regulated in both tissues at a given time point showed, that at 1 day after infection the hippocampus and the cortex share ~67% (1925 transcripts) of the regulated genes. At 3 days after infection the proportion of shared genes dropped to 52% in the cortex and 22% in the hippocampus (927 transcripts) (Figure 2B).

### Correspondence Analysis (COA)

**Figure 3:**
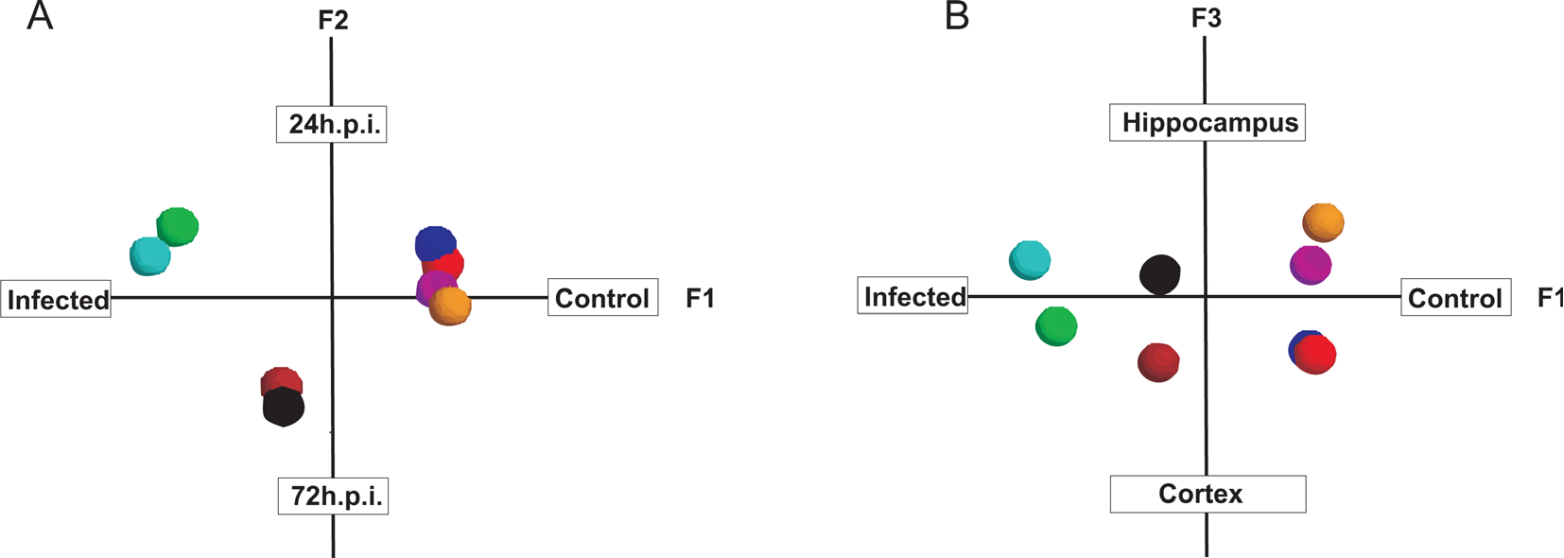
**COA analysis**. Correspondence Analysis based on the mean expression values (n = 3) of all genes that are expressed over background level in a given experimental group. Each experimental group is represented by a coloured globe. The 3 components with the highest explanatory value are visualized in two planes of projection: A [F1: health state, F2: time after infection] and B [F1: infection state, F3: tissue type].

Based on the Eigenvalues, which are a measure for the amount of data variance an Eigenvector describes, the 3 most informative components of the data can be defined. The 1st component (F1) which explains most of the variance in the data set (highest Eigenvalue) is the axis separating the non-infected controls from the infected animals. The second component (F2) separates the phases of the disease (1 day after infection/3 days after infection) and the third component (F3) discriminates between the investigated brain structures (cortex/hippocampus) (Figure 3 A/B). The identification of our experimental parameters (infection, time, tissue) with an unsupervised dimension reduction method confirms the validity of our dataset in regard to extract information about transcriptional regulation specific for a given experimental condition.

### GO statistics

The assessment of the gene expression profile in two differently affected brain structures at different time points allows extracting subpopulations of regulated genes which give insight into spatial and temporal mechanisms of the disease. In the presented study, statistics for the over-representation of biological processes was carried out for every tissue and time specific gene set, as well as for the intersection of all experimental endpoints.

### Genexpression in the hippocampus at 1 day after infection

From the 433 individual genes that were found to be significantly regulated in the hippocampus at 1 day after infection the most generic GO terms found to be significantly over-represented were cell differentiation (GO:0030154) and cell development (GO:0048731).

**Figure 4:**
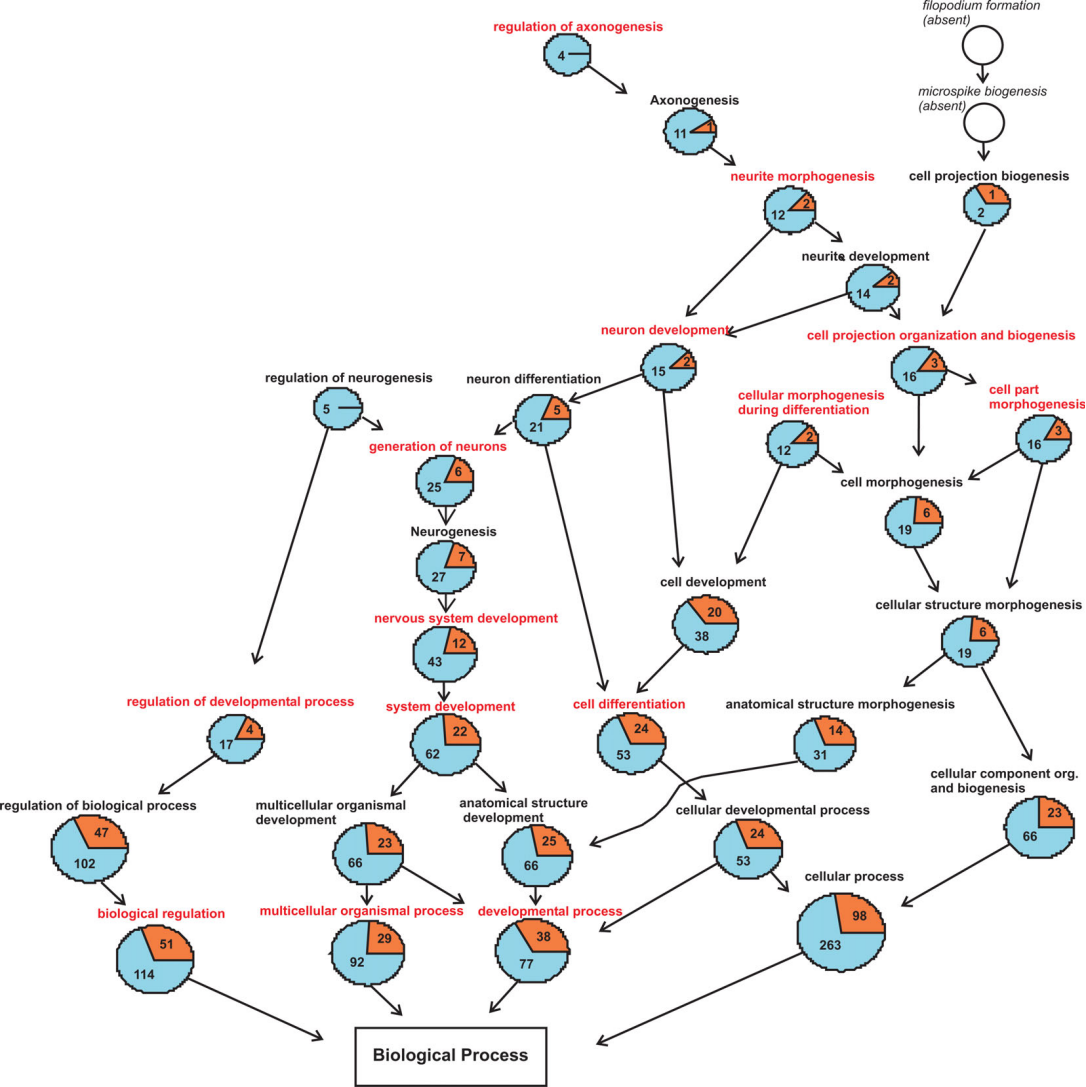
**GO graph hippocampus 1 day post infection**. GO graph induced by the biological processes which are over-represented in the gene set of the hippocampus at 1 day post infection. Specificity of the biological processes inclines from bottom to top of the graph. Compared to the totality of genes which are expressed in the hippocampus at 1 day after infection, the GO terms written in red are significantly over-represented in the collective of the significantly regulated genes in the hippocampus at 1 day after infection. Blue sections in pie charts illustrate the proportion of significantly down-regulated genes, red sections stand for significantly up-regulated genes. Strikingly, in all of the GO terms involved in developmental processes in general the significantly down-regulated genes are predominant.

Following the track of over-represented terms throughout the GO structure leads to processes involved in neuron generation and differentiation. Strikingly, the proportion of down-regulated genes is higher in all of the significantly over-represented processes providing evidence, that cellular development and differentiation is generally down-regulated in the hippocampus 1 day after infection (Figure 4). Analyzing the genes involved in neurogenic processes on a cellular pathway level shows, that key components of bone morphogenic proteins (BMPs) signaling are up-regulated (BMP2, Smad1, p300, STAT3, ID1). BMPs are members of the TGF-β family and are involved in the inhibition of neurogenesis by the induction of inhibitors of differentiation and DNA binding (IDs). The cellular components where these processes take place are the membrane, extracellular space and the proteinaceous extracellular matrix.

### Genexpression in the hippocampus at 3 days after infection

**Figure 5:**
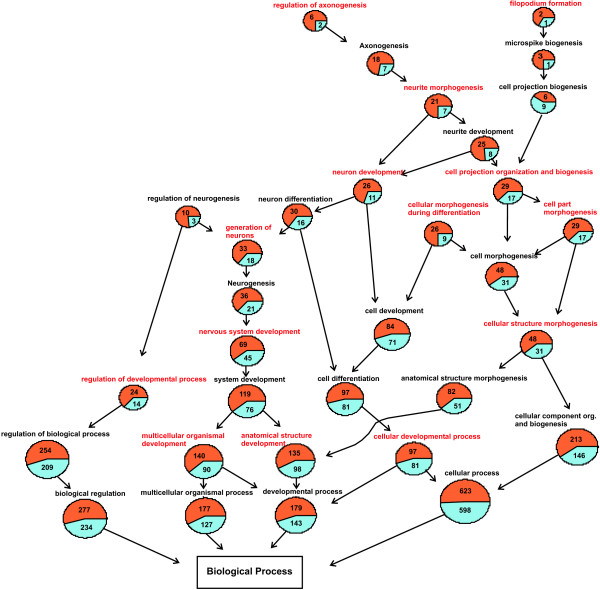
**GO graph hippocampus 3 days post infection**. GO graph induced by the biological processes which are over-represented in the gene set of the hippocampus at 3 days after infection. Specificity of the biological processes inclines from bottom to top of the graph. GO terms written in red are significantly over-represented in the significantly regulated gene set. The same GO terms describing neurogenic processes are over-represented as at 1 day after infection. Blue sections in pie charts illustrate the proportion of significantly down-regulated genes, red sections stand for significantly up-regulated genes. In contrast to 1 day post infection GO terms involved in developmental processes are represented mainly by significantly up-regulated genes

The GO graph for the visualization of the biological processes in the hippocampus at 3 days after infection is similar to the one at 1 day after infection due to the similarity of most of the significantly over-represented GO terms. The processes of nervous tissue development lead to processes of neuronal remodelling as there are positive regulation of neurogenesis (GO:0050769), regulation of axonogenesis (GO:0050770), filopodium formation (GO:0046847) and synapse organization and biogenesis (GO:0050808). In contrast to 1 day after infection, the majority of the genes involved in these processes are up-regulated (Figure 5).

**Figure 6:**
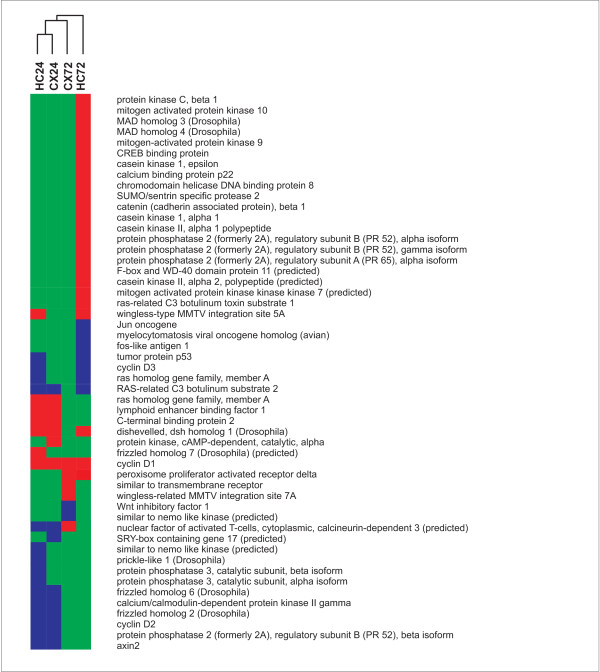
**Wnt signalling pathway**. Clustering of experimental groups based on significantly regulated genes of the wnt signalling pathway. Up-regulated [red] unchanged [green] down-regulated [blue]

**Figure 7:**
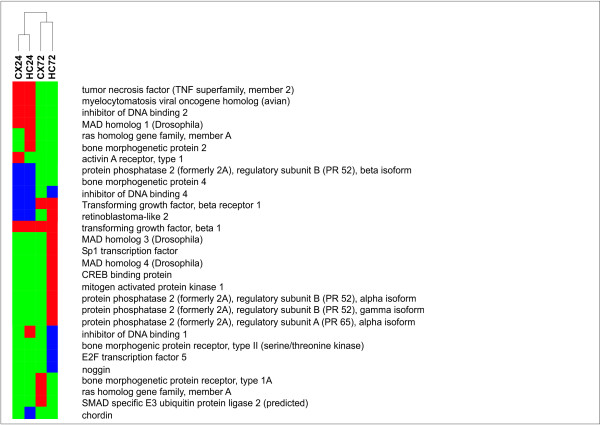
**TGF beta signalling pathway**. Clustering of experimental groups based on significantly regulated genes of the TGF-beta signalling pathway. Up-regulated [red] unchanged [green] down-regulated [blue]

The assignment of the transcripts found to be involved in neurogenic processes to KEGG biological pathways reveals, that a substantial number of genes are involved in the pro-neurogenic wnt and TGF-β signalling pathways (Figure 6, Figure 7). With a focus on the wnt signalling pathway we found an up-regulation of several genes involved in the canonical as well as non-canonical branch. For the canonical branch we found an up-regulation of components of the scaffolding complex which governs the phosphorylation and subsequent degradation of β-catenin. Wif-1, an inhibitor of the canonical wnt pathway was down-regulated and provides further evidence for pathway activation. The up-regulation of the wnt ligand wnt5a and the calcium binding protein p22 (CHP) indicates an activation of the non-canonical Wnt/Ca2+ branch.

Transcripts coding for the transforming growth factor beta 1 (TGF-β1) and its receptor TGF-β Receptor 1 as well as those coding for the receptor regulated R-Smads 2 and 3 and the Co Smad 4 were found to be up-regulated. In contrast to the acute phase of the disease (1 day after infection) the expression of transcripts involved in the anti-neurogenic BMP signalling pathway returned to control levels. Furthermore the expression of ID1 was significantly down-regulated. In parallel to the activation of pro-neurogenic pathways a significant increase in the rate of cell proliferation was observed in the dentate gyrus during the later phase of the disease (1-3 days after infection) compared to non infected control animals in the model used for the current study (infected:405 ± 25 BrdU cells/mm^2 ^, n = 4 vs non-infected:307 ± 51 BrdU cells/mm^2^, n = 5; unpaired t test p < 0.001) and in other models of pneumococcal meningitis [23,33].

### Genexpression in the cortex at 1 day after infection

In the cortex 378 annotated genes with a unique Entrez ID were found to be significantly regulated at 1 day after infection. In comparison to the other gene sets there were few and rather unspecific biological processes over-represented. Furthermore the analysis of the constructed GO graph reveals that the identified processes are solely connected through very generic parent terms. These findings imply that besides the processes of inflammation and immune response affecting both tissues at 1 day after infection there are no distinct disease mechanisms in progress in the cortical tissue at this early stage of the disease.

### Genexpression in the cortex at 3 days after infection

At 3 days after infection 421 individual annotated genes were found to be significantly expressed in the cortical tissue. The biological process cell communication (GO:0007154) was the most general GO term found to be significantly over-represented. The branches deriving from this term are ending in nodes involved in neurotransmitter transport, -signalling and -secretion (GO:0006836, GO:0007218, GO:0007269) as well as in processes of synaptic transmission (GO:0007268, GO:0035249, GO:0019226). As to be expected these processes mainly take place in the plasma membrane (synapse, dendrite, lammelopodium, growth cone) and vesicular system (clathrin coated vesicles) of the cells. Concerning the molecular function most of the genes fall into the categories of calcium ion binding, gated channel activity and transmembrane receptor activity.

### Time and tissue Intersection

The 322 individual genes which were significantly regulated at both 1 day after infection and 3 days after infection are mainly involved in processes of the immune- and inflammatory response.

The not antigen specific innate immune response is the first line of host defence against an invading pathogen. In accordance with the published literature [34] we found, that the Toll like receptor (TLR) signalling pathway is a predominant mechanism in PM for the initiation of inflammatory and immune genes. Transcripts coding for the pattern recognition receptors TLR2 and CD14 (mCD14), the adaptor protein MyD88 [17,35] and the nuclear factors I-kappaB and NF-kappaB as well as p38 and AP-1 were found to be up-regulated at both time points.

The transcripts coding for the heterodimer S100A8/S100A9 which are members of the damage-associated molecular pattern (DAMP) molecules show the highest expression value in infected animals of all transcripts detected on the chip. Upon stress S100A8/S100A9 are released into the extracellular compartment by damaged cells or macrophages and act as danger signals activating immune cells and vascular endothelium. As a consequence of the activation of the above described pathways, we found an up-regulation of mRNAs coding for cytokines, chemokines and their receptors as well as for components of the complement system.

Transcripts coding for proteins involved in the complement classical pathway (C1q, C1r, C1s, SERPING1), the lectin pathway (MASP1/2) and the alternative pathway (C3, HF1, BF) were found to be highly up-regulated.

**Figure 8:**
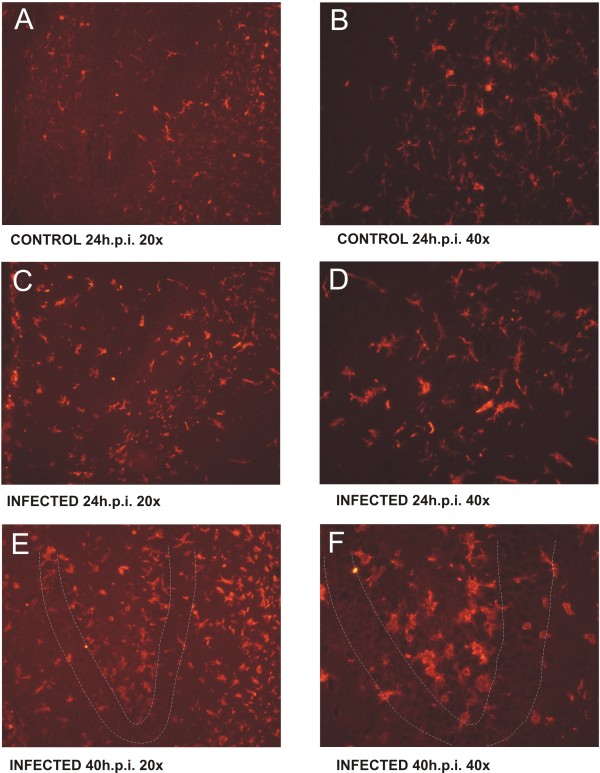
**Histomorphometry of microglia response**. The picture sequence demonstrates the morphological and spatial changes of microglia in response to experimental bacterial meningitis due to infection with *S .pneumoniae*. Microglial staining was performed by staining AIF-1 and sections were observed using a 20 × (pictures A, C and E), respectively 40 × (pictures B, D and F) magnifying objective. The resting microglia (picture A and B) transforms from a ramified to an amoeboid, phagocytic phenotype during the early acute phase of the disease (pictures C and D). Three days after infection we find a pronounced infiltration of microglial cells into the subgranular zone of the dentate gyrus (highlighted by dotted lines) where neuronal apoptosis occurs (picture E and F). The transformation and redistribution of the resting microglia to the site of tissue damage is in well agreement with the transcriptional data shown in figure 9.

**Figure 9:**
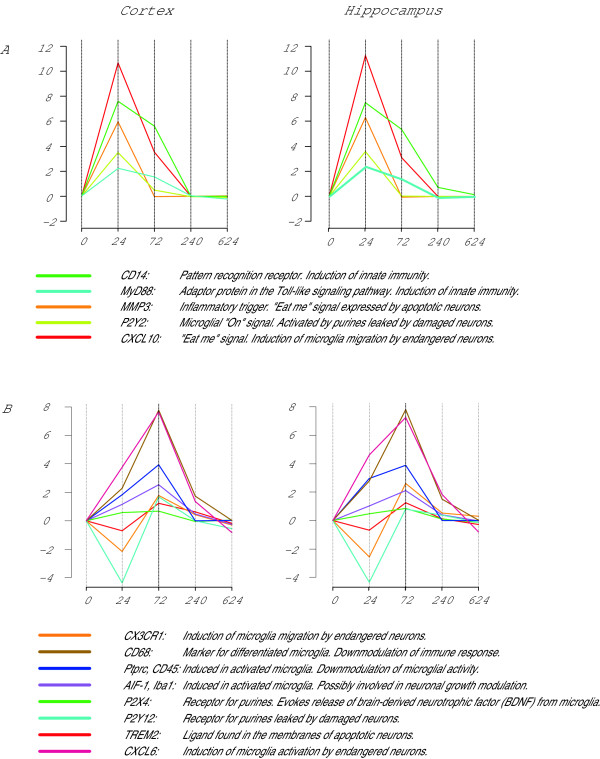
**Regulation of microglial response**. Expression kinetics of genes, involved in microglia regulation, in response to pneumococcal infection. A) Components of the Toll-like receptor pathway initiating the innate immune response, pro-inflammatory mediators and microglial "On" signals show their expression maxima in the acute phase of the disease at 1 day post infection (hpi). B) At 3 days after infection we find an expressional peak of transcripts coding for receptors that guide the resting microglia to the site of action and which orchestrate phagocytic and trophic functions during tissue regeneration. Furthermore there is a transcriptional peak of factors expressed in activated microglia which lead to a down-modulation of the immune response.

Concomitantly to the activation of immune and inflammatory pathways, the microglia specific allograft inflammatory factor 1 (AIF-1) was significantly up-regulated at 1 day after infection with a peak at 3 days after infection This regulation kinetics is in well accordance to the immunohistochemical detection of AIF-1, showing the distribution of microglia in brain tissue at 1 day after infection and 3 days after infection (Figure 8). A similar expression profile is observed for the macrophage specific marker CD68, which shows a pronounced up-regulation at 3 days after infection and is a correlative for the differentiation of microglia into mature macrophages. A morphological sign for microglia differentiation is the transformation from a ramified into a rod shaped and amoeboid form (Figure 8). Besides of these data confirming the histological findings, we found several transcripts which are involved in the orchestration of the microglial response to infection (Figure 9).

## Discussion

The evaluation of gene expression data is a challenging task due to statistical as well as interpretational pitfalls. Due to the complexity of the cellular networks the interaction between the different components is not known, and therefore a basic assumption of parametric hypothesis testing namely that the variables are independent and identical distributed is not met. The large number of features to be assessed per sample demands multiple testing corrections to minimize the number of false positives. Furthermore the often low sample number leads to inaccurate estimations of statistical parameters (e.g. the within group variability for a linear model or the mean value for fold-change calculation). As a consequence, the strategy of judging the biological relevance of a single transcript solely based on its fold-change or significant p-value is prone to be hampered by random effects.

The advent of biological databases governing a rapidly growing pool of gene annotation data permits to set gene expression profiles in a global biological context and thus putting newly drawn hypothesis on a more solid foundation.

In our work the relevance of our findings was optimized by the consideration of the following aspects. Due to the limited number of samples i.e. 3 animals per time point and tissue, the control of sample variability is crucial. As the main sources of variability we defined variations in the occurrence and extent of the disease-induced brain damage and the quality of the microarray probe generation. Standardization of brain damage was achieved by selecting animals with a similar score of apoptotic cells in the hippocampal dentate gyrus. Experimental *in vivo *models of infection inherently show a variability in clinical severity of disease and the corresponding disease parameters e.g. pathogen burden, inflammatory cyto-/and chemokines, and tissue destruction. Inter-individual variability in disease and the subsequent readouts (i.e. apoptosis) occurs also in the present experimental model of BM. Therefore we regard this kind of standardisation as mandatory also with larger sample groups to avoid an increase in gene expression variability, which may hamper the detection of minor but still biological significant expressional changes. RNA quality was assessed prior and after chip hybridization. After background correction and normalization of the raw data using a non-linear and robust multi array approach, the structure of the expression data was investigated by Correspondence Analysis (COA). The COA analysis revealed that all samples were separated correctly in regard to infection, sampling time and tissue type based on the inherent structure of the expression data. This finding indicates that there are substantial differences in the regulation of the transcriptome in regard to sampling time and sample origin, and demonstrates that the within-group-variability was sufficiently low to allow the separation of the groups (Figure 3).

After these pre-considerations, significance testing was performed with a moderated Bayesian t-statistics comparing each sample of the infected animals with the according age matched uninfected controls. Using age match controls is mandatory in order to eliminate genes that are differently expressed due to brain developmental processes, which may be ongoing in the experimental infant rat model used.

A further issue that has to be addressed in cerebral gene expression analysis is the complexity of the samples in regard to the different cell types within a given brain structure. This implies that the expressional pattern obtained from a given experimental condition is an overlay collage of various transcriptomic profiles from different cell populations. As a consequence the expressional level of a transcript depends on the extent of transcriptional regulation and the proportion of cells expressing the transcript. Therefore significant expressional changes in genes expressed only in a small fraction of cells may be diluted by the expressional "noise" by cells of the surrounding tissue. Furthermore reciprocal gene regulation in two cell populations can lead to an underestimation of real changes. Under this consideration the assignment of expressional changes to the evoking cell population is crucial for data interpretation.

In our study we focused on the events that take place in the acute and regenerative phase of bacterial meningitis in two distinct brain structures, namely the cortex and the hippocampus. In order to bring expressional data into a cell morphological context, we assessed the presence and distribution of microglia and that of proliferating cells in the hippocampus. These two cell populations play a pivotal role in the two disease phases investigated. There is growing evidence that the microglia takes the directorial role in coordinating the inflammatory response in the central nervous system (CNS) due to its capability to rapidly release autocrine and paracrine factors. Upon infection the microglia initiates a robust innate immune response in the immune-privileged brain tissue and is involved in the clean up of dying cells by the differentiation into a macrophage phenotype. Recent studies showed a strong correlation between the microglia activation induced by the pathogen and the modulation of hippocampal neurogenesis. Furthermore there is evidence, that the mode of microglia activation is an important determinant of neurogenesis and neuronal survival [22,23,33]. Whereas endotoxin activation leads to an impairment of neurogenesis and oligodendrogenesis, microglia activation by an adaptive immune response supports neuronal survival [36]. The interpretation of the expressional data in the following part of the discussion will be set in the light of the interaction between the activated microglia and the proliferating neuronal progenitor cells.

### Regulation of the host defence: Tissue and time Intersection

In the acute phase of the disease the genes that were found to be significantly expressed in both tissues were almost exclusively involved in processes of the immune- and inflammatory response. Many transcripts found to be regulated in the processes of the host defence have a function in the activation and propagation of the innate immunity response, the main defence system in the otherwise immune-privileged brain tissue.

The up-regulation of genes coding for pattern recognition receptors specific for Gram-positive bacteria (TLR2, CD14) expressed on astrocytes, oligodendrocytes and microglia indicates the recognition of invading *S.pneumoniae *by resident cells of the CNS. The concomitant up-regulation of several components of the Toll-like signalling pathway (MyD88, I-kappaB, NF-kappaB, AP-1) induces the expression of pro-inflammatory cytokines and components of the complement system triggering the activation of glial cells [17,34-38].

Glial activation is reflected by the up-regulation of cell type specific markers as there are allograft inflammatory factor 1 (AIF-1) and CD68 for microglia and glial fibrillary acidic protein (GFAP) specific for astrocytes. The induction of astrocyte activation is also reflected by an up-regulation of BMP-2, STAT3 and p300 which are factors in the BMP signalling pathway. Upon BMP binding to its receptor Smad1 is phosphorylated and migrates to the nucleus. There it forms a transcriptional complex with STAT3 and the co-activator p300 on the promoter sequence of the astrocyte-specific genes such as the one for the glial fibrillary acidic protein (GFAP) thereby inducing astrocyte activation [37-40]. The transcription level of the oligodendrocytes marker cyclic nucleotide phosphodiesterase 1 (CNP-1) was unaltered suggesting a limited role of this cell population in the initiation of host defence.

The activation of the microglia is also evident based on the immunohistochemical detection of AIF-1 protein in the course of the disease which shows the emergence of rod shaped amoeboid cells from the resting population expressing a more ramified phenotype (Figure 8). There is ample evidence from experimental studies, that the activation of astrocytes and microglia has a pronounced impact on the surrounding neuronal tissue as for example the induction of blood brain barrier disruption, vasculitis and apoptotic as well as necrotic cell death. In PM microglia are considered to form a first line of defence against invading bacteria and play a role in the regulation of the inflammatory reaction in response to infection. Extensive microglial activation has been shown to contribute to tissue damage in PM [16,18]. In previous studies we used immunohistochemistry and histomorphology to document an increase in microglia activation with disease progression in PM [15,19]. Correspondingly, microglial activation has been demonstrated in a rat model of ischemic brain injury [41].

The over-representation of biological processes involved in cell death and blood vessel morphogenesis and the localization of theses events to the proteinaceous extracellular matrix reflects these events in our expressional data. The evaluation of these processes on gene level reveals a high accordance with the findings of us and others in the field of bacterial meningitis thus validating our experimental approach [38,42-46]. In addition to the confirmation of recent data, we found new aspects concerning the modulation of microglial activity by surrounding neurons that to our knowledge have not yet been reported for bacterial meningitis. Traditionally microglia activation was regarded as a stereotypic and graded process with devastating consequences for the surrounding neurons. Recent findings indicate that microglial cells are able to integrate various inputs from the surrounding milieu and thereby tune their response appropriately. Microglia activation in the healthy brain is controlled by "On" and "Off" signals [37]. "Off" signals are constitutively expressed in the brain and their disappearance results in microglia activation [40]. In contrast "On" signals are produced on demand to initiate a pro- or anti-inflammatory microglial response [39]. According to their occurrence "On" and "Off" signals are separated into a released or a membrane bound fraction. Our data show that concomitantly with the up-regulation of the microglia associated transcripts (CD68 and AIF-1), the released "On" signals matrix-metalloproteinase-3 (MMP-3) and CXCL10 are up-regulated in the acute phase (1 day after infection) of the disease, whereas the released "Off" signal CX3CL1 is down-regulated. In vitro studies have shown that CXCL10 is secreted by endangered neurons and directs microglia migration by binding to the microglial CXC3 receptor.

The release of MMP-3 was also attributed to neuronal cell degradation. Previous studies [47] showed that besides its involvement in the disruption of the blood brain barrier, MMP-3 is released by apoptotic neuronal cell lines and mediates the discharge of cytokines of contiguous microglial cells. Thus it has been hypothesized that MMP-3 signalling may trigger inflammatory reactions as well as a rapid phagocytosis of apoptotic neurons.

With the progression of the disease into the late phase (3 days after infection) the up-regulation of the marker CD68 reflects microglial differentiation into a macrophagic phenotype. This finding is also reflected by our immunohistochemical data where we see a further increase in the number of rod shaped amoeboid microglial cells. The transition of microglial function from triggering the innate immune response to the phagocytic clearance of cellular debris and inflammatory control is also reflected in the type of microglial regulators. In our data we found the up-regulation of microglial receptors activated by pro-phagocytotic "eat me" signals and transcripts which function as immune silencers.

The metabotropic P2-purinoreceptor P2Y12 is stimulated by ATP released by injured neurons and thereby guides the microglial cells to areas of tissue damage [48,49]. TREM2, a further microglial receptor found to be up-regulated is involved in the modulation of inflammation and phagocytosis. Knockdown of TREM2 in mice inhibited phagocytosis of apoptotic neurons and increased gene transcription of pro- inflammatory genes [50-52]. Although pointing to a down-regulation of inflammation is the enhanced expression of CD68 a member of the family of neuroimmune regulatory proteins (NIRegs). These proteins may play an important role in controlling lymphocyte/macrophage/microglia hyperinflammatory responses, while sparing host defence and repair mechanisms. Moreover, NIRegs have direct beneficial effects on neurogenesis and are contributing to brain tissue remodelling [53].

### Regenerative phase of the disease

While in the acute phase of the disease (1 day after infection) similar GO terms were over-represented in the hippocampal and the cortical set of regulated genes, the results of GO statistics in the late acute phase (3 days after infection) diverge. Whereas in the cortex processes of synaptic transmission were over-represented, GO terms associated with gene regulation at 3 days after infection in the hippocampus are pointing to processes of neurogenesis and neuronal migration. KEGG pathway analysis revealed an activation of the TGF-β and wnt signalling pathway, two pathways known to play a central role in different aspects of cell development.

The wnt signalling pathway is known to participate in several aspects of neural development including stem cell proliferation and maintenance, fate determination, axon guidance, dendrite development and synapse formation [54]. Upon binding of a member of the wnt family to the corresponding receptor two distinct pathways can be activated. The first pathway is the canonical branch which activates the β-catenin/TCF transcriptional regulatory complex, and the second one is the non-canonical branch, which involves a wide range of downstream signalling effectors.

The influence of the wnt signalling on the target cells is modulated by a complex regulatory network integrating various inputs including the level of ligand-receptor interactions and the diversity of downstream transcriptional complexes. In the adult brain, components of the canonical wnt signalling pathway were found to be expressed in both astrocytes and neuronal stem cells in neurogenic niches e.g. the subgranular zone (SGZ) of the dentate gyrus. Furthermore, an activation of canonical wnt/β-catenin/TCF was found in proliferating cells and a subset of newly generated neurons. Blockade of wnt signalling reduces the number of proliferating neuronal restricted precursors (NRPs) but had no effect on the general population of proliferating cells or glia production [39].

In the CNS, TGF-β is known as an injury-related cytokine produced by astrocytes and neurons [55-57]. The binding of TGF-β to the serine-threonine kinase receptors TGF-βRI/TGF-βRII activates the transcription factors Smad 2/3 and Smad 4. Upon complex formation the Smad 2/3-4 complex translocates to the nucleus and regulates the response to TGF-β signalling i.e. cell cycle control, regulation of early development and differentiation and neuron cell survival [56,58,59]. It has been recognized, that TGF-β secretion by astrocytes in the adult brain has a primarily neuroprotective role. One mode of action is the protection against N-methyl-D-aspartic acid (NMDA)-induced excitatory neuronal death by the TGF-β mediated reduction of the NMDA-evoked Ca+ influx [60]. An alternative explanation for a neuroprotective effect of TGF-β has been proposed by TGF-β mediated activation of nuclear factor- kappaB which in turns exerts an anti-apoptotic effect on neuronal cells [55].

The cross talk between the wnt- and TGF-β pathway is mediated by the cooperation between TCF/LEFs and the SMAD transcription factors that are bound to adjacent DNA elements, thereby allowing coordinated regulation of target genes [61,62].

In the present study the up-regulation of key components of the wnt- as well as the TGF-β signalling pathway is accompanied by the emergence of proliferating BrdU labeled cells in the subgranular zone (SGZ) of the dentate gyrus. Considering a central role for the canonical wnt signalling pathway in neuronal proliferation and fate commitment[39], and acknowledging the fact that this pathway is exclusively up-regulated in the hippocampus in the late phase of the disease, we conclude that the expression of wnt signalling components is evoked by the population of recently divided cells (BrdU-labeled) in the SGZ, and that a substantial part of these cells have a neuronal commitment. This notion is supported by recent studies [22,23,33,63] assessing neurogenesis in the SGZ after experimental PM which show that approximately 70% of the BrdU-labeled cells are positive for the neuronal markers TUC-4, MAP-2 and beta-tubulin. The, concomitant tissue- and time-specific activation of both, the wnt-signalling- and the TGF-β Smad2/Smad3-pathway, is indicative for a synergistic involvement of both pathways in the fine tuning of neuronal tissue regeneration beyond a merely neuroprotective function of TGF-β.

### Implications for therapeutic strategies

An important finding of the present study is that on a transcriptional level, the major events with respect to the regulation of the host response occur within the first 3 days after infection. Beyond this time, there were no major differences detectable in the gene expression patterns of infected- vs control animals. A strong activation of neurogenic processes in the hippocampal dentate gyrus was observed both by gene expression and immunohistochemical labeling of proliferating cells starting as early as 3 days after infection e. g. during the acute and sub acute phase of the disease. Thus, there is a therapeutic window of opportunity during the acute phase of bacterial meningitis for interventions aimed at supporting mechanisms of brain regeneration in bacterial meningitis.

Moreover, therapies that potentially impede the ongoing regenerative processes must be avoided at this time. In this context our findings add a new viewpoint to the discussion on the pros and cons of adjunctive dexamethasone treatment which is recommended to be initiated before or together with the first dose of antibiotic therapy and continued for four days [64]. While there are studies that demonstrated a beneficial effect of dexamethasone on the mortality in patients with pneumococcal meningitis, the benefit of the treatment for the reduction of neurological sequelaes is unproven [3,8,64,65]. In a more recent study a meta-analysis of individual patient data from the randomised, double-blind, placebo-controlled trials of dexamethasone for bacterial meningitis in patients of all ages for which raw data were available was performed. After analysis of the data from 2029 patients from five trials the authors concluded that adjunctive dexamethasone in the treatment of acute bacterial meningitis does not significantly reduce death or neurological disability[8]. Furthermore, there are a number of experimental studies showing that dexamethasone provokes an increase of apoptotic cells in the SGZ of the dentate gyrus [31,66,67], which leads to aggravated learning impairments in PM survivors [31]. In the light of the present data one may speculate that the observed learning impairments upon dexamethasone treatment may be due to an impediment of neurogenic processes in the hippocampus during the early regenerative phase of the disease. A putative target for such an impediment is the pro-neurogenic wnt signalling pathway which we found activated in the hippocampus at 3 days after infection. In vitro it has been shown, that glucocorticoids inhibit wnt signalling by activating glycogen synthase kinase 3 beta (GSK3β)[68] by inhibition of the TCF/LEF transcription factor [69] and by enhancing the expression of Wnt inhibitors [70-72]. Further emphasis on the importance of the wnt signalling pathway for hippocampal neurogenesis is provided by very recent studies showing, that the neurogenic effects of antidepressants is most likely effectuated by an activation of the wnt signal transduction. Wexler et al [73] showed that the mood stabilizer lithium enhances the pool of adult hippocampal progenitor cells and induces a neuronal cell fate. The authors attributed these effects to a lithium dependent wnt signalling induction by a down-regulation of GSK3β and an up-regulation of beta-catenin. In the same line of evidence it was demonstrated that the chronic administration of the dual reuptake inhibitor venlafaxine induces the nuclear translocation of beta-catenin most likely by an inhibition of GSK3β through AKT/PKB signalling [74]. Based on these findings antidepressants may have the potential to support hippocampal neuro-regenerative processes in the recovery phase of pneumococcal meningitis.

## Conclusions

The exponential growth of biological metadata resources within the last decade advances the analysis of datasets produced by massive parallel methodologies from the mere publication of expansive gene lists to a more meaningful biological systems based approach.

In this work these metadata resources were used to identify pivotal gene regulatory events of the animal host in response to pneumococcal meningitis. Besides the confirmation of recent findings by other researchers in the field this work expands the insight in the brains immunoregulative network as well as in neurogenic repair mechanisms. Based on the more detailed description of these processes therapeutic schemes can be developed which balance the benefits of anti-inflammatory drugs in the early phase of the disease and pro-neurogenic agents during regeneration.

## List of Abbreviations

AIF-1: allograft inflammatory factor 1; BM: bacterial meningitis; BMP: bone morphogenic proteins; BrdU: 5-bromo2-deoxyuridine; CNS: central nervous system; CSF: cerebrospinal fluid; CFU: colony forming units; COA: correspondence analysis; DAMP: damage-associated molecular pattern; DAPI: 4',6-Diamidino-2'-phenylindole; FU: fluorescent units; GO: gene ontology; GAPDH: glyceraldehyde-3-phosphate dehydrogenase; IRQ: interquarantile range; KEGG: Kyoto encyclopedia of genes and genomes; MMP-3: matrix-metalloproteinase-3; NIR: neuroimmune regulatory proteins; NMDA: N-methyl-D-aspartic acid; PFA: Paraformaldehyde; PBS: phosphate buffered saline; PM: pneumococcal meningitis; PCA: principal component analysis; SGZ: subgranular zone.

## Competing interests

The authors declare that they have no competing interests.

## Authors' contributions

MW contributed to the study design, performed the statistical data analysis and drafted the manuscript. DG carried out the immunohistochemistry and provided expertise in microglia regulation and neuronal apoptosis. JR assisted in the animal experiments and performed the microarray experiments as well as the immunoassays. SLL conceived and supervised the study, participated in its design and coordination, and contributed to writing the manuscript. All authors read and approved the final manuscript.

## Pre-publication history

The pre-publication history for this paper can be accessed here:

http://www.biomedcentral.com/1471-2334/10/176/prepub
